# Resistance to Antibiotics, Biocides, Preservatives and Metals in Bacteria Isolated from Seafoods: Co-Selection of Strains Resistant or Tolerant to Different Classes of Compounds

**DOI:** 10.3389/fmicb.2017.01650

**Published:** 2017-08-31

**Authors:** José L. Romero, María J. Grande Burgos, Rubén Pérez-Pulido, Antonio Gálvez, Rosario Lucas

**Affiliations:** Microbiology Division, Department of Health Sciences, University of Jaen Jaen, Spain

**Keywords:** antimicrobial resistance, biocides, antibiotics, metals, seafood

## Abstract

Multi-drug resistant bacteria (particularly those producing extended-spectrum β-lactamases) have become a major health concern. The continued exposure to antibiotics, biocides, chemical preservatives, and metals in different settings such as the food chain or in the environment may result in development of multiple resistance or co-resistance. The aim of the present study was to determine multiple resistances (biocides, antibiotics, chemical preservatives, phenolic compounds, and metals) in bacterial isolates from seafoods. A 75.86% of the 87 isolates studied were resistant to at least one antibiotic or one biocide, and 6.90% were multiply resistant to at least three biocides and at least three antibiotics. Significant (*P* < 0.05) moderate or strong positive correlations were detected between tolerances to biocides, between antibiotics, and between antibiotics with biocides and other antimicrobials. A sub-set of 30 isolates selected according to antimicrobial resistance profile and food type were identified by 16S rDNA sequencing and tested for copper and zinc tolerance. Then, the genetic determinants for biocide and metal tolerance and antibiotic resistance were investigated. The selected isolates were identified as *Pseudomonas* (63.33%), *Acinetobacter* (13.33%), *Aeromonas* (13.33%), *Shewanella, Proteus* and *Listeria* (one isolate each). Antibiotic resistance determinants detected included *sul1* (43.33% of tested isolates), *sul2* (6.66%), *bla*_TEM_ (16.66%), *bla*_CTX−M_ (16.66%), *bla*_PSE_ (10.00%), *bla*_IMP_ (3.33%), *bla*_NDM−1_ (3.33%), *floR* (16.66%), *aadA1* (20.0%), and *aac(6*′*)-Ib* (16.66%). The only biocide resistance determinant detected among the selected isolates was q*acE*Δ*1* (10.00%). A 23.30 of the selected isolates were able to grow on media containing 32 mM copper sulfate, and 46.60% on 8 mM zinc chloride. The metal resistance genes *pcoA/copA, pcoR*, and *chrB* were detected in 36.66, 6.66, and 13.33% of selected isolates, respectively. Twelve isolates tested positive for both metal and antibiotic resistance genes, including one isolate positive for the carbapenemase gene *bla*_NDM−1_ and for *pcoA/copA*. These results suggest that exposure to metals could co-select for antibiotic resistance and also highlight the potential of bacteria on seafoods to be involved in the transmission of antimicrobial resistance genes.

## Introduction

The global world fish market reached 175 million tons in 2016 (Food and Agriculture Organization (FAO), 2016). There is a growing concern in the fisheries sector about the increasing prevalence of multidrug-resistant bacterial strains in the food chain (EFSA, 2010; ECDC, 2012, 2013; EFSA-ECDC, 2013; Watts et al., 2017). Multidrug-resistant bacteria carrying extended-spectrum (ESBL) β-lactamase genes can now be found in healthy humans as well as in various animal species, food and even in environmental samples, and have become a threat in hospital settings. Carbapenemase-producing *Enterobacteriaceae* are particularly of concern because they tend to spread, making infection treatment difficult (Iovleva and Doi, 2017). Resistance genes for antimicrobials such as β-lactams, quinolones, and fluoroquinolones may be associated with plasmids. Plasmids of fluoroquinolone resistance in *Enterobacteriaceae* are widespread, and are often associated with resistance to β-lactams in strains with multidrug resistant phenotypes (Crémet et al., 2011). Some genes, such as the variant *aac(6*′*)-Ib-cr* simultaneously confer resistance to aminoglycosides, and fluoroquinolones (Kim et al., 2011). Others like the efflux pump *oqxAB* described in *Escherichia coli*, confer resistance to various antibiotics and biocides (Hansen et al., 2007; Wong and Chen, 2013).

Different types of selective pressure (such as antibiotics, biocides, or heavy metals) could play a role in the prevalence of antimicrobial resistance in the food chain. Biocides may co-select strains resistant to antibiotics of clinical use, as verified in the case of triclosan, and others (Chuanchuen et al., 2001; Braoudaki and Hilton, 2004; Ortega-Morente et al., 2013). Biocides are widely used in disinfecting equipment and facilities, including fish farming, and fish processing facilities (Directive 98/8/CE). In Gram-negative bacteria, several genes for resistance to biocides belonging to the group of quaternary ammonium compounds (QACs) as *qacE, qacE*Δ*1, qacF, qacG*, and *qacH* have been described. Among them, *qacE*Δ*1* is the most widespread, as it is found in various groups of Gram-negative bacteria (Kücken et al., 2000). Association of *qacE*Δ*1* (and less frequently also *qacH* and *qacF*) with class I integrons together with antibiotic resistance genes has been reported (Mulvey et al., 2006; De Toro et al., 2011). A study of microbial populations in environments contaminated with QACs related the increased incidence of resistance to QACs with a higher incidence of class I integrons (Gaze et al., 2005), suggesting that exposure to QACs could also selected for antibiotic resistance associated with such integrons. Other antimicrobials such as lysozyme-EDTA combinations and chemical preservatives such as sodium lactate and trisodium phosphate also deserve attention because of their wide use and potential applications in the food industry for decontamination and food preservation (Lucera et al., 2012; Bjornsdottir-Butler et al., 2015; Wang et al., 2015). There is also a growing interest in extending the use of plant essential oils or their antimicrobial compounds (such as carvacrol or thymol) for disinfection and food preservation (Lucera et al., 2012; Patel, 2015; Wang et al., 2015). One study showed that exposure to pine oil induced a decreased susceptibility to a range of antimicrobial compounds (including antibiotics and biocides; Moken et al., 1997). Yet, little is known about development of resistance/tolerance to these compounds, and co-resistance to antibiotics.

Metal salts are used for decontamination in fish farming. Metals such as copper (Cu) and zinc (Zn) are essential micronutrients in living things, but they can become toxic if they are above a certain concentration. Copper and zinc are frequently used in aquaculture and also as antifouling paints on boats (Yebra et al., 2004; Watermann et al., 2005; Guardiola et al., 2012). Copper can be released into the environment by both human activities and natural processes. Zinc is rarely found in nature in its metallic state, but many minerals contain zinc as a main component. The main anthropogenic sources of zinc are mining, zinc production facilities, iron, and steel production, corrosion of galvanized structures, coal, and fuel combustion, waste disposal and incineration, and the use of fertilizers, and pesticides containing zinc (WHO, 2001). Fish can accumulate and transmit heavy metals along the food chain. The genes that control resistance to metals may be associated with plasmids, which provide bacteria a competitive advantage over other organisms when specific metals are present (Trevors et al., 1985; Hobman and Crossman, 2014). The appearance of multidrug resistance plasmids carrying resistance to heavy metals is alarming and requires additional monitoring (Gómez-Sanz et al., 2013). The co-location of metal-resistance and antimicrobial-resistance genes can facilitate their persistence, co-selection, and dissemination (Gómez-Sanz et al., 2013; Wales and Davies, 2015).

The aim of this study was to provide insights on resistance to clinically relevant antibiotics in bacterial strains isolated from seafood sold at supermarkets and fishmarket. Since exposure to other types of antimicrobials such as biocides and metals could co-select for antibiotic resistance, we hypothesized that antibiotic-resistant strains from seafoods could also be phenotypically resistant, and/or carry genetic determinants of resistance to other antimicrobials, thus increasing the risk of transmission of antibiotic resistance through the seafood production chain. Therefore, susceptibility of bacterial strains isolated from different seafood samples against various biocides, antibiotics, and metals was evaluated, and the presence of resistance genes in multiresistant strains was determined. A special emphasis was made on extended-spectrum β-lactamase genes because of the risks that ESBL-producing bacteria may pose to human health.

## Materials and methods

### Bacterial isolation

A total of 22 seafood samples from 16 different fish and seafood species purchased at supermarkets and fishmarket in the province of Jaen (Spain) during the years 2013 and 2014 were investigated (Table 1). Unless indicated, samples consisted of unprocessed whole specimens from sea fishing, and were sold over the counter on ice. Samples (25 g each) were suspended in 225 ml of buffered peptone water supplemented with 10 g of NaCl/liter and homogenized by stomaching with as Stomacher 400 (Sewald, UK). The samples were analyzed following standard procedures for microbiological analysis of food by plating serial dilutions on Trypticase Soya Agar (TSA, Scharlab, Barcelona, Spain) supplemented with 10 g of NaCl/liter (saline TSA, STSA). After 24–48 h incubation at 30°C, colonies were purified by streaking on TSA (without added NaCl), and the pure cultures were examined by Gram-staining, catalase and oxidase tests, and stored at −80°C in Trypticase Soya Broth (TSB, Scharlab) supplemented with 20% glycerol.

**Table 1:** Sensitivity to antibiotics, biocides and other antimicrobials in bacterial isolates recovered from seafoods.

**Food source and isolate**	**Viable counts (Log_10_ CFU/g)**	**RESISTANCE PHENOTYPE**	**BC**	**CT**	**HDP**	**TC**	**CF**	**CHX**	**PHMG**	**OX**	**CVC**	**THY**	**SL**	**TSP**	**L**	**LE**
Anchovy (*Engraulis encrasicholus*)	4.27; 3.80; 4.32															
**B2**		**AMP, CTX, CM, NA, TM/STX, TC**	**25**	**7.5**	**25**	>**250**	**25**	**5**	**25**	**25**	**0.050**	**0.250**	**5.00**	**1.00**	>**0.01**	**ABC**
B6			25	2.5	25	25	5	20	2.5	7.5	0.010	0.010	3.00	0.75	>0.01	ABC
**B7**		**AMP, CM, NA, TM/STX**	**25**	**7.5**	**50**	**5**	**25**	**5**	**5**	**2.5**	**0.050**	**0.250**	**5.00**	**0.75**	>**0.01**	**ABC**
B22			25	2.5	25	25	25	15	5	25	0.010	0.010	3.00	0.75	>0.01	ABC
B24			25	2.5	25	25	25	20	5	10	0.010	0.010	3.00	0.75	>0.01	ABC
**4B1**		**AMP, NA, TM/STX**	**25**	**2.5**	**25**	**5**	**25**	**10**	**25**	**25**	**0.100**	**0.010**	**5.00**	**0.75**	>**0.01**	**ABC**
4B21		AMP, TM/STX	25	2.5	25	5	25	10	25	25	0.100	0.050	5.00	0.75	>0.01	ABC
4B22		NA, CHX	25	0.5	25	25	5	50	7.5	10	0.010	0.010	3.00	1.00	>0.01	ABC
4B23		AMP, NA, CHX	25	2.5	25	25	25	50	7.5	10	0.100	0.125	5.00	0.75	>0.01	ABC
**4B31**		**AMP, CTX, IMP, CM, TM/STX, BC, CT, HDP, PHMG, TC**	**250**	**25**	>**250**	>**250**	**25**	**20**	**100**	**25**	**1.00**	**0.125**	**5.00**	**1.00**	>**0.01**	**ABC**
4B41		NA	25	0.5	25	5	5	1	5	25	0.010	0.010	3.00	1.00	>0.01	ABC
**4B43**		**AMP, CTX, CM, NA, TM/STX**	**25**	**2.5**	**50**	**5**	**25**	**10**	**25**	**25**	**1.000**	**0.125**	**5.00**	**0.75**	>**0.01**	**ABC**
Sardine (*Sardina pilchardus*)	4.63; 4.50															
**4Sd1**		**AMP, NA, TM/STX, CHX, OX**	**25**	**5**	**25**	**50**	**25**	**50**	**25**	>**250**	**0.100**	**0.050**	**5.00**	**1.00**	>**0.01**	**ABC**
4Sd3		BC	250	2.5	25	5	5	5	2.5	7.5	0.100	0.010	3.00	0.50	>0.01	ABC
4Sd21		CHX, PHMG, OX	25	5	25	50	25	50	100	>250	0.050	0.010	5.00	1.50	>0.01	C
4Sd22		TM/STX, TC	50	7.5	25	>250	25	15	25	75	0.100	0.050	5.00	0.75	>0.01	ABC
**4Sd41**		**AMP, CTX, CM, S, CHX, PHMG, TC**	**50**	**7.5**	**25**	>**250**	**25**	**50**	**100**	**250**	**0.100**	**0.250**	**5.00**	**0.75**	>**0.01**	**ABC**
**4Sd42**		**AMP, NA, TM/STX**	**25**	**2.5**	**25**	**5**	**25**	**5**	**25**	**100**	**0.050**	**0.125**	**5.00**	**0.50**	>**0.01**	**ABC**
4Sd51		AMP, S	50	0.5	25	50	25	10	5	250	0.050	0.010	5.00	1.50	>0.01	ABC
4Sd52		AMP	50	2.5	25	50	25	20	7.5	250	0.025	0.010	5.00	0.75	>0.01	ABC
**4Sd53**		**AMP, CHX**	**25**	**2.5**	**25**	**25**	**25**	**50**	**25**	**250**	**0.100**	**0.250**	**3.00**	**1.00**	>**0.01**	**ABC**
4Sd54			50	0.5	25	5	5	1	5	100	0.025	0.010	1.00	0.25	>0.01	ABC
Blue whiting (*Micromesistius poutassou*)	3.78															
4Ba1		AMP, TM/STX	25	2.5	25	5	25	10	7.5	250	0.050	0.250	3.00	0.50	>0.01	ABC
4Ba2			5	0.5	5	5	5	1	0.5	5	0.025	0.010	3.00	1.00	>0.01	ABC
4Ba31		AMP, TM/STX	5	0.5	5	5	5	1	5	50	0.010	0.010	1.00	0.25	>0.01	ABC
4Ba32			5	0.5	5	5	5	1	0.5	5	0.010	0.010	1.00	0.25	>0.01	ABC
4Ba33		IMP	25	2.5	5	25	25	15	7.5	250	0.010	0.250	3.00	0.50	>0.01	ABC
4Ba41			25	2.5	25	25	25	15	5	50	0.025	0.250	5.00	0.75	>0.01	ABC
4Ba42		AMP, TM/STX	25	2.5	25	5	25	10	7.5	50	0.025	0.010	3.00	0.50	>0.01	ABC
Athlantic horse mackerel (*Trachurus trachurus*)	6.25															
**J2**		**AMP, CTX, CM, S, NA, TM/STX, CT, HDP, TC**	**5**	**25**	>**250**	**250**	**25**	**10**	**0.5**	**5**	**0.100**	**0.250**	**5.00**	**1.00**	>**0.01**	**ABC**
**J4**		**AMP, CTX, CM, TM/STX, BC, CT, HDP, TC**	>**250**	**25**	>**250**	**250**	**25**	**10**	**25**	**100**	**0.100**	**0.250**	**3.00**	**1.00**	>**0.01**	**ABC**
J5		NA	5	0.5	5	5	5	1	0.5	0.5	0.025	0.250	5.00	1.50	>0.01	ABC
J6		CM	25	2.5	50	25	25	5	5	25	0.010	0.010	3.00	1.00	>0.01	ABC
**J7**		**AMP, S, BC**	**250**	**7.5**	**50**	**25**	**25**	**5**	**2.5**	**75**	**0.100**	**0.125**	**3.00**	**0.75**	>**0.01**	**ABC**
Mackerel (*Scomber scombrus*)	4.93															
**Cb22**		**AMP, IMP, CHX**	**25**	**0.5**	**25**	**50**	**25**	**50**	**10**	**250**	**0.025**	**0.250**	**5.00**	**3.00**	>**0.01**	**ABC**
**Cb27**		**AMP, CTX, IMP, CM, NA, TM/STX, TC**	**50**	**7.5**	**25**	**250**	**25**	**10**	**25**	**250**	**0.050**	**0.250**	**5.00**	**1.50**	>**0.01**	**ABC**
**Cb212**		**AMP, CTX, CM, NA, TM/STX, TC**	**50**	**7.5**	**25**	>**250**	**25**	**10**	**25**	**250**	**0.100**	**0.250**	**5.00**	**1.50**	>**0.01**	**ABC**
Sole (*Solea* spp.)	4.99; 5.62															
Le1		AMP	5	2.5	25	25	25	20	0.5	5	0.010	0.010	3.00	0.75	>0.01	ABC
Le2			25	2.5	50	50	25	15	5	75	0.010	0.010	3.00	0.75	>0.01	ABC
Le4		AMP	25	2.5	50	50	25	15	5	75	0.010	0.010	3.00	0.75	>0.01	ABC
Le5		CHX	25	2.5	50	50	25	50	5	50	0.010	0.010	3.00	0.75	>0.01	ABC
Hake (*Merluccius capensis*; sliced)	5.84															
M135		CHX	5	0.5	25	5	5	50	0.5	5	0.010	0.010	3.00	1.00	>0.01	ABC
Blue shark (*Prionace glauca*; sliced)	3.62															
T213			25	2.5	25	5	25	5	2.5	50	0.025	0.010	3.00	1.00	0.0025	ABC
**T215**		**S, CHX**	**50**	**7.5**	**50**	**50**	**25**	**50**	**25**	**250**	**0.025**	**0.010**	**5.00**	**1.50**	>**0.01**	**ABC**
Gilthead seabream (*Sparus aurata*)	6.44															
**Do11**		**AMP, IMP, CHX**	**25**	**2.5**	**25**	**25**	**25**	**50**	**7.5**	**250**	**0.025**	**0.250**	**3.00**	**3.00**	>**0.01**	**ABC**
Do15			25	2.5	50	50	25	20	10	250	0.010	0.010	3.00	1.00	>0.01	ABC
Do24		AMP, IMP, CHX	25	2.5	25	50	25	50	5	100	0.025	0.010	5.00	3.00	>0.01	ABC
Do26		S, CHX	25	2.5	50	50	25	50	25	75	0.010	0.010	3.00	1.00	>0.01	ABC
Sea bass (*Dicentrarchus labrax*; aquaculture)	5.88; 5.70															
Lbi14		CHX	25	2.5	50	50	25	50	25	50	0.010	0.010	3.00	0.75	>0.01	ABC
**Lbi15**		**AMP, CTX, IMP, CM, NA, TM/STX, TC**	**50**	**7.5**	**50**	>**250**	**25**	**20**	**25**	**100**	**0.100**	**0.250**	**5.00**	**1.50**	>**0.01**	**ABC**
**Lbi16**		**AMP, CTX, CAZ, IMP, CM, S, NET, CIP, NA, TM/STX, PHMG, TC, OX**	**75**	**5**	**50**	**250**	**25**	**10**	**100**	>**250**	**0.100**	**0.250**	**3.00**	**1.00**	>**0.01**	**ABC**
**Lbi25**		**AMP, CTX, IMP, CM, S, NA, TM/STX, BC, CT, HDP, PHMG, TC, OX**	>**250**	**25**	**250**	**250**	**25**	**10**	**100**	>**250**	**0.100**	**0.250**	**3.00**	**0.25**	>**0.01**	**ABC**
Salmon (*Salmo salar*; slices packed in trays; aquaculture)	7.85; 7.47															
S11		AMP, CTX, CAZ, CM, NA	25	2.5	25	5	25	10	25	250	0.010	0.025	5.00	1.00	0.0025	ABC
S12		AMP, CTX, CM, S	25	2.5	25	5	25	10	25	250	0.010	0.010	3.00	1.00	0.0025	ABC
S13		AMP, CAZ, CM	25	2.5	25	5	25	20	25	250	0.010	0.010	3.00	0.75	0.0025	ABC
**S14**		**AMP, CAZ, CM, S, NA**	**25**	**2.5**	**25**	**5**	**25**	**20**	**25**	**250**	**0.010**	**0.025**	**3.00**	**1.00**	**0.0025**	**ABC**
S15		AMP, CTX, CM, NA	25	2.5	25	5	25	20	25	250	0.010	0.025	3.00	1.00	0.0025	ABC
S16		AMP, CTX, CM, TE	25	2.5	25	5	25	15	25	250	0.010	0.025	3.00	1.00	0.0025	ABC
S21		AMP, CM, NA	25	2.5	25	5	25	10	25	250	0.010	0.025	3.00	1.00	0.0025	ABC
**S22**		**AMP, CTX, CM, S, NA**	**25**	**2.5**	**25**	**5**	**25**	**20**	**25**	**250**	**0.010**	**0.025**	**3.00**	**1.00**	**0.0025**	**ABC**
S23		AMP, CTX, CM, NA	25	2.5	25	5	25	10	25	250	0.010	0.025	5.00	1.50	0.0025	ABC
S24		AMP, CTX, CM, NA	25	2.5	25	5	25	10	25	250	0.010	0.025	5.00	1.50	0.0025	ABC
**S25**		**AMP, CTX, CM, S, NA**	**25**	**2.5**	**25**	**5**	**25**	**10**	**25**	**250**	**0.010**	**0.025**	**3.00**	**1.50**	**0.0025**	**ABC**
S26		AMP, CM, NA	25	2.5	25	5	25	10	25	250	0.010	0.025	3.00	1.50	0.0025	ABC
Squid (*Loligo* spp.)	4.44															
C121			25	2.5	25	25	25	5	5	25	0.025	0.010	3.00	1.50	>0.01	ABC
C123			25	2.5	25	25	25	5	2.5	25	0.025	0.010	3.00	1.00	>0.01	ABC
C125			25	2.5	25	25	25	5	2.5	25	0.025	0.010	3.00	1.00	>0.01	ABC
C126			25	2.5	25	25	25	5	5	25	0.025	0.010	3.00	1.00	>0.01	ABC
Juvenile squid (*Loligo vulgaris*)	4.49															
4C1		CAZ, S, NA	25	0.5	25	25	5	5	5	250	0.025	0.010	1.00	0.75	>0.01	ABC
4C4		AMP, TM/STX	25	2.5	5	5	5	1	0.5	5	0.025	0.010	1.00	0.25	>0.01	ABC
**4C21**		**CTX, CAZ, S, NA**	**25**	**2.5**	**25**	**25**	**5**	**5**	**5**	**250**	**0.025**	**0.010**	**1.00**	**0.75**	>**0.01**	**ABC**
4C22		AMP, TM/STX	25	5	25	5	25	10	7.5	75	0.100	0.250	3.00	0.50	>0.01	ABC
4C23		CTX, CAZ, S, NA	25	2.5	25	5	5	5	5	100	0.025	0.010	1.00	0.75	>0.01	ABC
4C31			25	2.5	25	25	25	15	7.5	250	0.025	0.250	3.00	0.50	>0.01	ABC
**4C32**		**AMP, CTX, IMP, CM, NA, BC, CT, HDP, CHX, PHMG, TC**	>**250**	**25**	>**250**	>**250**	**25**	**50**	**100**	**250**	**0.100**	**0.125**	**5.00**	**1.00**	>**0.01**	**ABC**
4C51		CAZ, S	5	0.5	5	5	5	1	5	5	0.010	0.010	1.00	0.25	>0.01	ABC
**4C52**		**AMP, CTX, C, NA, TM/STX, HDP, TC**	**75**	**10**	**250**	>**250**	**25**	**15**	**25**	**250**	**0.100**	**0.010**	**5.00**	**0.75**	>**0.01**	**ABC**
Mussels (*Mytilus edulis*)	3.65															
**Mj2**		**AMP, IMP, S, CHX**	**25**	**5**	**25**	**50**	**25**	**50**	**7.5**	**250**	**0.025**	**0.250**	**5.00**	**3.00**	>**0.01**	**ABC**
Prawns (*Penaeus* spp.; boiled and frozen; aquaculture)	4.60															
L122		AMP, CAZ	25	2.5	25	25	25	5	5	50	0.025	0.010	3.00	1.00	>0.01	ABC
**L123**		**AMP, CTX, CAZ, IMP, CM, S, NET, TE, CIP, TM/STX**	**25**	**2.5**	**25**	**5**	**25**	**5**	**0.5**	**50**	**0.010**	**0.010**	**3.00**	**1.00**	>**0.01**	**ABC**
**L124**		**AMP, IMP, CHX**	**25**	**5**	**25**	**50**	**25**	**50**	**10**	**250**	**0.025**	**0.250**	**5.00**	**3.00**	>**0.01**	**ABC**
L125			25	2.5	25	5	5	5	2.5	**250**	0.010	0.250	3.00	1.00	>0.01	ABC
Norway lobster (*Nephrops norvergicus*; boiled and frozen)	4.47															
Cg11			25	2.5	25	5	5	5	2.5	250	0.010	0.010	3.00	3.00	>0.01	ABC
Cg12			5	2.5	25	5	5	5	0.5	5	0.010	0.010	3.00	1.50	>0.01	ABC
Cg14			25	2.5	25	5	25	5	2.5	25	0.010	0.010	3.00	1.00	>0.01	ABC
Cg21			25	2.5	25	5	5	5	2.5	250	0.025	0.010	3.00	3.00	>0.01	ABC
**Cg22**		**AMP, CM, NA, TM/STX, TC**	**25**	**5**	**25**	**250**	**25**	**5**	**25**	**250**	**0.050**	**0.250**	**5.00**	**1.50**	>**0.01**	**ABC**

*In bold, isolates selected for further study*.

*AMP, ampicillin; CTX, cefotaxime, CAZ, ceftazidime; IMP, imipenen; CIP, ciprofloxacin; NA, nalidixic acid; CM, chloramphenicol; S, streptomycin; NET, netilmicin; TE, tetracycline; TM/STX, trimethoprim/solfamethoxazole. The minimum inhibitory concentrations (MICs) for biocides (in mg/l, or in microliters of commercial solution per liter in the case of OX), phenolic compounds and preservatives (in %) are indicated. BC, benzalkonium chloride; CT, cetrimide; CHX, chlorhexidine; HDP, hexadecylpyridinium chloride; TC, triclosan; PHMG, polyhexamethylen guanidium chloride; OX, P3 Oxonia (in microliters of commercial solution per; CVC, carvacrol; THY, thymol (THY); SL, sodium lactate; TSP, trisodium phosphate; L, lysozyme (L). LE, lysozyme-EDTA combinations (A, B, C indicate the different LE inhibitory combinations)*.

### Determination of resistance to antibiotics, biocides, and other antimicrobial compounds

A collection of 87 bacteria randomly isolated from the different seafood samples were screened for sensitivity to biocides, antibiotics, and other antimicrobial compounds as described below. Benzalkonium chloride (BC), cetrimide (CT), hexadecylpyridinium chloride (HDP), chlorhexidine digluconate (CHX), triclosan (TC), and hexachlorophene (CF) were from Sigma-Aldrich (Madrid, Spain). The commercial solution of benzalkonium chloride contained 50% (wt/v) of the active compound. Triclosan and hexachlorophene were dissolved (10% wt/v) in 96% ethanol. HDP (5% wt/v), CT (10% wt/v), and CHX (20% wt/v) were dissolved aseptically in sterile distilled water. Poly-(hexamethylen guanidinium) hydrochloride (PHMG) solution (containing 7.8% of PHMG, by weight) was a kind gift of Oy Soft Protector Ltd (Espoo, Finland). P3 oxonia (OX, containing 25–35% hydrogen peroxide, 0.83–2.5 N acetic acid, and 0.26–0.66 N peracetic acid) was supplied by ECOLAB (Barcelona, Spain). Biocide solutions were stored at 4°C for ≤7 days. Carvacrol (CVC), thymol (THY), sodium lactate (SL), trisodium phosphate (TSP), lysozyme (L), and ethylenediaminetetraacetic acid (EDTA) were from Sigma-Aldrich. Solutions containing 100 mg/l lysozyme and 5 mM EDTA were combined in different proportions to yield the following final concentrations: A, 30 mg/l lysozyme plus 3.5 mM EDTA; B, 50 mg/l lysozyme plus 2.5 mM EDTA; C, 70 mg/l lysozyme plus 1.5 mM EDTA. Minimum inhibitory concentrations (MIC's) were determined by the broth microdilution method on 96-well, flat-bottom microtiter plates (Becton Dickinson Labware, Franklin Lakes, NJ). Briefly, serial dilutions of each antimicrobial were inoculated (1%, vol/vol) with overnight cultures of bacterial strains grown in Trypticase Soya Broth (TSB; Scharlab). Growth and sterility controls were included for each isolate. Microtiter plates were incubated at 30°C. Optical density (OD 595 nm) readings were performed with an iMark Microplate Reader (BioRad, Madrid) after 24–48 h incubation. All assays were done in triplicate.

Antibiotic resistance was determined by the disk diffusion method as described by the Clinical and Laboratory Standards Institute CLSI (2015) on cation-adjusted Mueller-Hinton agar (Fluka, Sigma-Aldrich, Madrid, Spain). Disks containing ampicillin (AMP, 10 μg), ceftazidine (CAZ, 30 μg), cefotaxime (CTX, 30 μg), imipenem (IMP, 10 μg), streptomycin (S, 10 μg), tetracycline (TE, 30 μg), ciprofloxacin (CIP, 5 μg), nalidixic acid (NA, 30 μg), and trimethoprim/sulfamethoxazole (TM/STX, 1.25/23.75 μg) were supplied by Biomérieux (Madrid, Spain). Chloramphenicol (CM, 30 μg) was from BBL (Madrid, Spain).

### Identification of antimicrobial-tolerant isolates

From the preliminary screening on antimicrobial resistance, 30 isolates were selected for further study based on food source, antibiotic resistance and biocide tolerance. Selected isolates were resistant to at least three antibiotics or at least to one antibiotic and one biocide. The 30 isolates were identified by 16S rDNA sequencing. DNA was extracted with a bacterial genomic DNA extraction kit (GenEluteTM, Sigma-Aldrich) and 16 S rDNA was amplified as described by Abriouel et al. (2005). PCR amplification products were purified using a GFX PCR DNA and Gel Band Purification Kit (GE-Healthcare, Spain), and then sequenced according to Weisburg et al. (1991) in a CEQ 2000 XL DNA Analysis System (Beckman Coulter, CA, USA). The DNA sequences obtained were searched for homology by using the BLAST algorithm available at the National Center for Biotechnology Information (NCBI, USA).

### Determination of tolerance to metals

The selected 30 isolates were tested for tolerance to copper and zinc metals as follows. Mueller–Hinton II agar plates (Sigma-Aldrich, Madrid, Spain) were supplemented with CuSO_4_•5H_2_O (PanReac, Barcelona, Spain) (4, 8, 12, 16, 20, 24, 32, and 36 mM, adjusted to pH 7.2) or ZnCl_2_ (PanReac) (2, 4, 8, and 16 mM, adjusted to pH 6.5) according to Cavaco et al. (2011). Then, plates were inoculated with 2 μl from overnight cultures of bacterial strains diluted 10-folds in sterile saline solution. The plates were incubated at 37°C under aerobic conditions and inspected for bacterial growth after 24 h. The lowest metal concentration that inhibited growth of the inoculated bacterial strains was taken as the MIC.

### Investigation of genetic determinants of resistance

The selected 30 isolates were investigated for the presence of genetic determinants of resistance. The presence of the biocide resistance genes *qacE* and *qacE*Δ*1* and their possible association with class I integrons was investigated by PCR according to Chuanchuen et al. (2007). Specifically, the forward primer qacEF was used in combination with reverse primers qacER and sulR for amplification of *qacE, qacE*Δ*1* and the 3′ coding sequence (3′ CS).

The following extended-spectrum β-lactamase genetic determinants were investigated by PCR: *bla*_TEM_ (Sáenz et al., 2004), *bla*_PSE_ (Chiu et al., 2006), *bla*_CTX−M_, and *bla*_CTX−M−2_ (Bertrand et al., 2006), as well as carbapenemases *bla*_IMP_, *bla*_*NDM*−1_, *bla*_OXA−23_, and *bla*_VIM−2_ (Ramakrishnan et al., 2014). Other antimicrobial resistance genes investigated by PCR were the aminoglycoside resistance genes *aadA1* (Guerra et al., 2001), and *aac(6*′*)-Ib* (Park et al., 2006), the phenicol resistance genes *floR* (Chiu et al., 2006) and *cmlA* (Sáenz et al., 2004), and the sulfonamide and trimethoprim resistance genes *sul1* (Guerra et al., 2001), *sul2* (Sáenz et al., 2004), and *sul3* (Sáenz et al., 2010).

The gene for the multicopper oxidase found in the plasmid-borne operons *pcoABCDRSE* (described in *Pseudomonas syringae*) and *copABCDRS* (*E. coli*) was investigated using the primers and PCR conditions described for *pcoA*/*copA* by Badar et al. (2014). The additional genes *copB, copC, copD* (Kamika and Momba, 2013; Badar et al., 2014) and *pcoR* (Brown et al., 1992) were also investigated. The chromosomal *cueAR* operon, encoding a putative P1-type ATPase and a MerR-type regulatory protein involved in copper homeostasis in *Pseudomonas putida* was investigated according to Adaikkalam and Swarup (2002). The *czcD* gene involved in the regulation of the CZC zinc, cobalt, and cadmium efflux system was investigated by PCR according to Medardus et al. (2014). The chromate resistance gene *chrB* was investigated as described by Chihomvu et al. (2015) and Nies et al. (1990).

### Statistical analysis

The relationships between resistances to the different antimicrobials tested were studied by Principal component analysis with Pearson correlation coefficient (r) by using IBM SPSS Statistics 22 (IBM Corporation, Armonk, New York, USA) and Mystat statistics and graphics package (Systat Software, Hounslow, London, UK; evaluation version 2015.1). Positive correlations were defined as very weak (0.00–0.19), weak (0.20–0.39), moderate (0.4–0.59), strong (0.60–0.79) or very strong (0.80–0.99), with a *P* significance of <0.05.

## Results

### Microbial load and bacterial isolation

The microbial load (aerobic mesophiles) of the different seafood samples is shown in Table 1. Most samples had viable cell counts comprised between 4 and 7 log CFU/g. Lowest viable counts were reported for blue shark, and highest counts were found in refrigerated raw salmon slices packed in trays.

After viable cell counting, bacterial colonies grown on saline TSA from highest dilutions were repurified by streaking on TSA without added salt. This was done so in order to avoid possible interference of added salt in growth media with antimicrobial resistance tests. A total of 87 bacterial colonies isolated at random were selected, representing the different seafood products sampled (Table 1). Eighty two of them were Gram-negatives (including mainly bacilli), while the remaining five were Gram-positive (including four cocci and one rod).

### Antimicrobial resistance

The 87 isolates were tested for sensitivity to antibiotics, biocides and other antimicrobials (cavacrol, thymol, sodium lactate, trisodium phosphate, lysozyme, and different lysozyme-EDTA combinations; Table 1). The incidence of resistance to β-lactam antibiotics was highest for AMP (57.47% of isolates), followed by CTX (27.59%), IMP (14.94%), and CAZ (11.49%). Resistance to protein synthesis inhibitors was detected mostly for CM (33.33%) and S (20.69%), while resistance to TE (2.30%) and NET (2.30%) was low. Remarkably, a 35.63% of isolates were resistant to NA, but only 2.30% were resistant to CIP. A 27.58% of isolates were resistant to TM/STX. A 39.08% of isolates were resistant to three or more antibiotics, and 18.40% were resistant to five or more. Two isolates were resistant to 10 out of the 11 antibiotics tested.

Bacterial isolates were tested for biocide tolerance in two groups (Gram-positives, and Gram-negatives) since Gram-negative bacteria in general have greater tolerance to biocides because of the outer membrane permeability barrier. Only low percentages of the Gram negative isolates showed high tolerance levels to the biocides BC (5.75%; MIC ≥ 250 mg/l), CT (5.75%; MIC ≥ 25 mg/l), HDP (6.90%; MIC ≥ 250 mg/l), PHMG (6.90%; MIC ≥ 100 mg/l), and OX (4.60%; MIC > 250 μl/l) (Table 1). Higher percentages of biocide-tolerant isolates were obtained for CHX (19.54%; MIC ≥ 50 mg/l) and TC (16.09%; MIC ≥ 250 mg/l). Among the Gram positive isolates, only one had a high MIC of 250 mg/l for BC. A total of seven isolates showed high tolerance to three or more biocides (Table 1).

Isolates showed large differences in sensitivities to carvacrol and thymol (Table 1). Only two isolates had MICs higher than 0.25% for carvacrol, and 16.39% of isolates required 0.1% for inhibition. By contrast, 43.68% of isolates were inhibited by very low carvacrol concentration (0.01%). Regarding thymol, 26.44% of isolates had a MIC of 0.25%, but none required concentrations higher than this value for inhibition. There was also a high percentage of isolates (55.17%) that were inhibited by a low concentration of thymol (0.025%).

Most isolates were inhibited by sodium lactate at 3% (55.17%) or 5% (35.63%), while the rest (9.20%) were inhibited at 1% (Table 1). However, isolates were more heterogeneous in sensitivity to trisodium phosphate (TSP). About 80% of isolates were inhibited at TSP concentrations in the range of 0.75–1.5%, and only a small percentage (8.04%) required a TSP concentration of 3% for inhibition.

Most isolates (85.06%) were resistant to the highest concentration of lysozyme tested (100 mg/l), and only a low percentage (14.94%) were inhibited by a low lysozyme concentration (2.5 mg/l; Table 1). When lysozyme was tested in combination with EDTA at different proportions, all combinations were effective against most isolates, except for one isolate that only was inhibited by lysozyme-EDTA combination C containing a higher proportion of lysozyme to EDTA.

### Correlations between antimicrobial resistances

**Figure 1:**
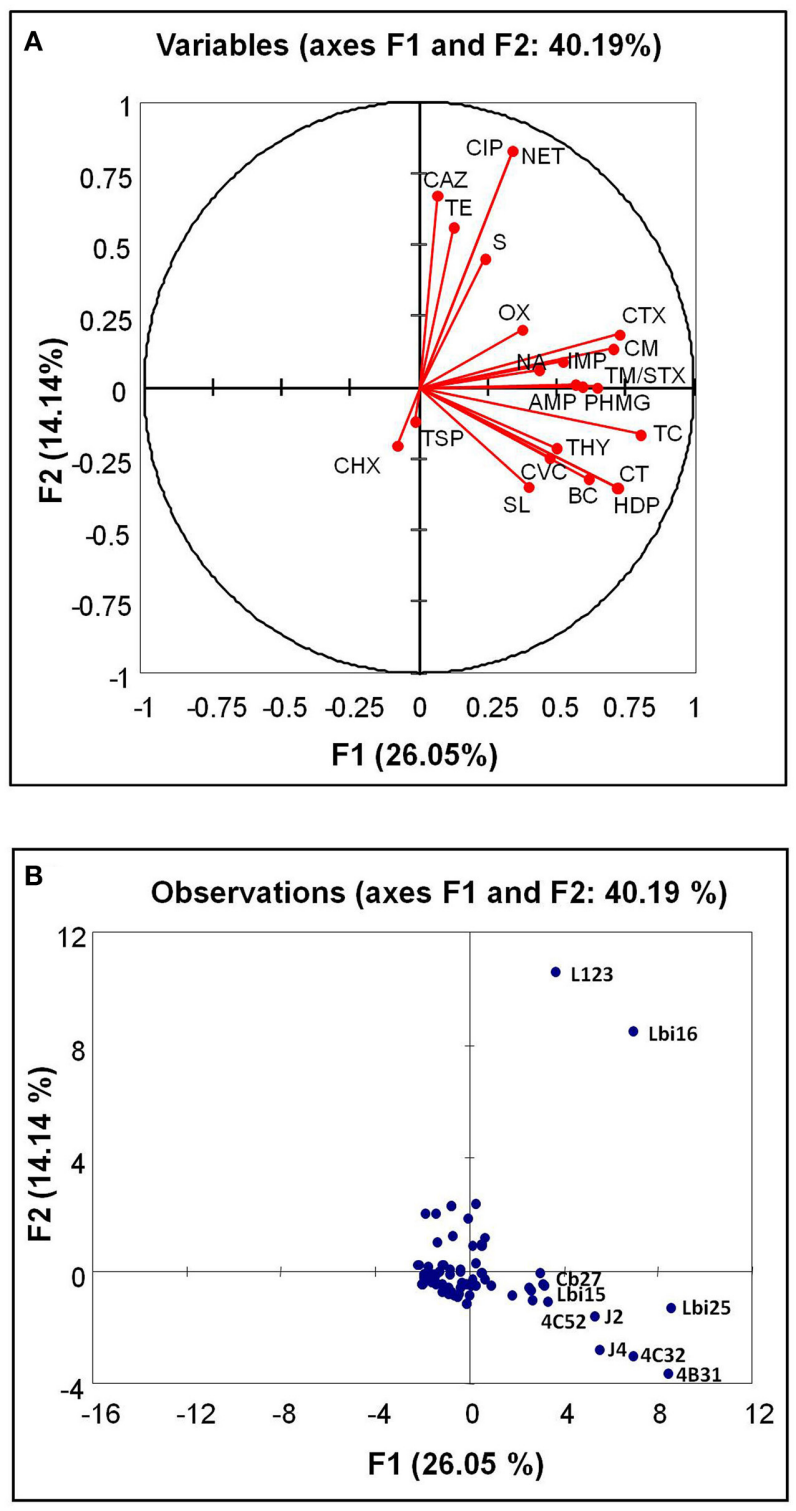
Biplot for biocide tolerance and antimicrobial resistance (scores) in the 87 bacterial isolates (variables) from seafoods. Antimicrobials **(A)**, red dots, and isolates **(B)**, blue dots are indicated. In **(B)**, the letters indicate the bacterial isolates with an outstanding high number of antimicrobial resistance traits. BC, benzalkonium chloride; CT, cetrimide; HDP, hexadecylpyridinium chloride; TC, triclosan; CF, hexachlorophene; PHMG, poly-(hexamethylen guanidinium) hydrochloride; OX, P3 oxonia; AMP, ampicillin; CTX, cefotaxime; CAZ, ceftazidime; IMP, imipenem; CM, chloramphenicol; S, streptomycin; TE, tetracycline; NA, nalidixic acid; TM/STX, trimethoprim-sulfamethoxazole; CVC, carvacrol; THY, thymol; SL, sodium lactate; TSP, trisodium phosphate.

Of the 87 isolates, 66 (75.86%) were resistant to antibiotics and/or tolerant to biocides. Comparing biocides and antibiotics, of the 29 isolates tolerant to at least one biocide (33.33%), 27 (31.03%) were also resistant to at least one antibiotic (Table 1). Six isolates (6.90%) were tolerant to at least three biocides and at least three antibiotics. The correlations between the different antimicrobials tested for the 87 bacterial isolates are shown in Figure 1 and Table 2. Only the statistically significant (*P* < 0.05) positive correlations that were moderate, strong or very strong are described below. The statistically significant (*P* < 0.05) weak correlations are also listed in Table 2, but are not described in the text.

**Table 2:** Correlations between antibiotic resistance and tolerance to biocides and other antimicrobials in the 87 bacterial isolates recovered from seafood.

**Variables**	**BC**	**CT**	**CHX**	**HDP**	**TC**	**PHMG**	**OX**	**AMP**	**CTX**	**CAZ**	**IMP**	**CIP**	**NA**	**CM**	**S**	**NET**	**TE**	**TM/ STX**	**CVC**	**THY**	**SL**	**TSP**
**BC**	**1.000**																					
**CT**	**0.788**	**1.000**																				
**CHX**	0.003	0.003	**1.000**																			
**HDP**	**0.712**	**0.907**	−0.020	**1.000**																		
**TC**	**0.429**	**0.564**	−0.137	**0.621**	**1.000**																	
**PHMG**	**0.517**	**0.517**	0.095	**0.463**	**0.498**	**1.000**																
**OX**	0.182	0.182	0.169	0.157	0.203	**0.590**	**1.000**															
**AMP**	**0.212**	**0.212**	−0.045	**0.234**	**0.313**	0.142	0.078	**1.000**														
**CTX**	**0.290**	**0.400**	−**0.239**	**0.441**	**0.570**	**0.339**	0.110	**0.427**	**1.000**													
**CAZ**	−0.089	−0.089	−0.178	−0.098	−0.060	0.044	0.093	0.018	0.181	**1.000**												
**IMP**	**0.312**	**0.312**	**0.363**	**0.268**	**0.343**	**0.395**	**0.216**	**0.295**	**0.246**	0.051	**1.000**											
**CIP**	−0.038	−0.038	−0.076	−0.042	0.142	**0.261**	**0.333**	0.132	**0.249**	**0.426**	**0.366**	**1.000**										
**NA**	0.023	0.126	−0.125	0.176	**0.327**	0.082	0.180	**0.349**	**0.507**	0.183	0.025	0.046	**1.000**									
**CM**	**0.244**	**0.349**	−**0.287**	**0.385**	**0.553**	**0.289**	0.078	**0.559**	**0.764**	0.127	0.182	**0.217**	**0.543**	**1.000**								
**S**	0.118	0.118	−0.037	0.085	0.085	0.197	0.159	0.095	**0.320**	**0.439**	0.104	**0.300**	0.153	0.181	**1.000**							
**NET**	−0.038	−0.038	−0.076	−0.042	0.142	**0.261**	**0.333**	0.132	**0.249**	**0.426**	**0.366**	**1.000**	0.046	**0.217**	**0.300**	**1.000**						
**TE**	−0.038	−0.038	−0.076	−0.042	−0.067	−0.042	−0.034	0.132	**0.249**	0.185	0.151	**0.488**	−0.114	**0.217**	0.111	**0.488**	**1.000**					
**TM/STX**	0.179	**0.290**	−**0.239**	**0.339**	**0.570**	0.136	**0.233**	**0.479**	**0.310**	−0.061	0.174	**0.249**	**0.293**	**0.327**	−0.061	**0.249**	0.077	**1.000**				
**CVC**	**0.368**	**0.368**	−0.068	**0.350**	**0.279**	**0.335**	0.042	**0.216**	**0.298**	−0.083	0.179	−0.004	0.101	**0.250**	−0.061	−0.004	−0.051	**0.373**	**1.000**			
**THY**	**0.216**	**0.276**	0.125	**0.221**	**0.493**	**0.221**	0.118	**0.341**	0.205	−0.166	**0.444**	0.068	0.169	**0.230**	0.005	0.068	−0.099	**0.388**	0.167	**1.000**		
**SL**	0.054	0.135	**0.212**	0.178	**0.371**	0.178	0.084	**0.408**	0.195	−**0.331**	**0.239**	−0.066	**0.265**	**0.291**	−0.173	−0.066	−0.066	**0.278**	**0.273**	**0.388**	**1.000**	
**TSP**	−0.107	−0.088	**0.368**	−0.104	−0.032	−0.069	−0.049	0.109	−0.039	−0.142	**0.426**	−0.019	−0.049	−0.015	−0.032	−0.019	−0.019	−**0.225**	−0.077	0.171	**0.358**	**1.000**

*BC, benzalkonium chloride; CT, cetrimide; CHX, chlorhexidine; HDP, hexadecylpyridinium chloride; TC, triclosan; PHMG, polyhexamethylen guanidium chloride; OX, P3 Oxonia; AMP, ampicillin; CTX, cefotaxime, CAZ, ceftazidime; IMP, imipenen; CIP, ciprofloxacin; NA, nalidixic acid; CM, chloramphenicol; S, streptomycin; NET, netilmicin; TE, tetracyclin; TM/STX, trimethoprim/solfamethoxazole; CVC, carvacrol; THY, thymol; SL, sodium lactate; TSP, trisodium phosphate*.

*Data in bold indicate significant correlations at P < 0.05 level*.

The following pairs of biocides showed positive correlations that were very strong (CT-HDP), strong (BC-CT, BC-HDP, and HDP-TC) or moderate (BC-TC, BC-PHMG, CT-TC, CT-PHMG, HDP-PHMG, TC-PHMG, and PHMG-OX).

For antibiotics, the following positive correlations were very strong (CIP-NET), strong (CTX-CM) or moderate (AMP-CTX, AMP-CM, AMP-TM/STX, CTX-NA, CAZ-CIP, CAZ-SM, CAZ-NET, CIP-TE, NA-CM, and TE-NET).

For the rest of antimicrobials tested (CVC, THY, SL, TSP), the only significant (*P* < 0.05) correlations detected were weak (Table 2).

Interestingly, significant (*P* < 0.05) positive correlations were also detected between antimicrobials belonging to the different groups tested. For example, moderate positive correlations were detected for the biocides CT, HDP, and TC with the antibiotic CTX, and also for TC with CM and TM/STX and with the phenolic compound THY (Table 2). A few antibiotics showed moderate positive correlations with the rest of antimicrobials tested, as in the case of AMP with SL and IMP with THY and TSP.

From the preliminary general study, 30 isolates were selected for further analysis regarding metal resistance, identification, and study of the genetic determinants of resistance.

### Identification of selected isolates

The 30 isolates selected for further study were identified by 16s rDNA sequencing (Table 3). Most of them (96.66%) were identified as Gram negative bacteria: *Pseudomonas brassicacearum* (10.00%), *Pseudomonas poae* (16.67%), *P. putida* (3.33%), *Pseudomonas synxantha* (26.67%), *Pseudomonas* spp. (6.67%), *Acinetobacter calcoaceticus* (10.00%), *Acinetobacter oleivorans* (3.33%), *Aeromonas salmonicida* (10.00%), *Aeromonas* spp. (3.33%), *Shewanella baltica* (3.33%), and *Proteus mirabilis* (3.33%). The only Gram positive isolate identified belonged to *Listeria innocua* (3.33%).

**Table 3:** Characterization of selected isolates.

**Isolate**	**Food source**	**Genetics determinants of resistance**	**CuSO_4_ (MIC)**	**ZnCl_2_ (MIC)**
*Pseudomonas brassicacearum* 4Sd1	Sardine	*sul1, chrB*	16	4
*Pseudomonas brassicacearum* 4B1	Anchovy	*chrB*	16	4
*Pseudomonas brassicacearum* 4B43	Anchovy		16	4
*Pseudomonas poae* B2	Anchovy	*sul1, floR*	20	16
*Pseudomonas poae* J7	Athlantic horse mackerel	*floR, pcoR, chrB*	32	8
*Pseudomonas poae* 4Sd41	Sardine		36	16
*Pseudomonas poae* 4Sd42	Sardine	*bla*_PSE_, *aadA1, pcoR, chrB*	12	4
*Pseudomonas poae* 4C52	Squid	*bla*_CTX−M_, *aadA1, pcoA/copA*	32	4
*Pseudomonas putida* Cg22	Norway lobster	*bla*_TEM_, *pcoA/copA*	32	16
*Pseudomonas synxantha* J2	Athlantic horse mackerel	*sul1, bla*_CTX−M_	36	16
*Pseudomonas synxantha* J4	Athlantic horse mackerel	*sul1, floR, pcoA/copA*	20	16
*Pseudomonas synxantha* Cb27	Mackerel	*aac(6′)-Ib, pcoA/copA*	36	16
*Pseudomonas synxantha* Lbi15	Sea bass	*aadA1, pcoA/copA*	20	8
*Pseudomonas synxantha* Lbi16	Sea bass	*pcoA/copA*	24	8
*Pseudomonas synxantha* Lbi25	Sea bass	*sul1, sul2, floR, aadA1, pcoA/copA*	24	16
*Pseudomonas synxantha* 4C32	Squid	*sul1, bla*_PSE_, *bla*_CTX−M_, *pcoA/copA*	36	16
*Pseudomonas synxantha* 4B31	Anchovy	*bla*_NDM−1_, *pcoA/copA*	36	16
*Pseudomonas* spp. B7	Anchovy	*sul1, sul2, floR, bla*_IMP_, *aadA1, pcoA/copA*	36	8
*Pseudomonas* spp. Cb212	Mackerel	*pcoA/copA*	36	16
*Shewanella baltica* 4Sd53	Sardine	*sul1, aac(6′)-Ib*	16	4
*Acinetobacter calcoaceticus* S14	Salmon	*qacEΔ1, sul1, bla*_TEM_, *aac(6′)-Ib*	32	16
*Acinetobacter calcoaceticus* S22	Salmon	*qacEΔ1, sul1, bla*_TEM_, *aac(6')-Ib*	32	16
*Acinetobacter calcoaceticus* S25	Salmon	*qacEΔ1, sul1, aac(6′)-Ib*	32	16
*Acinetobacter oleivorans* L123	Prawn	*bla*_TEM_, *bla*_CTX−M_	24	4
*Aeromonas salmonicida* Cb22	Mackerel	*sul1, bla*_TEM_	20	8
*Aeromonas salmonicida* Mj2	Mussel		12	8
*Aeromonas salmonicida* L124	Shrimp		32	8
*Aeromonas* spp. Do11	Gilthead seabream	*sul1, bla*_TEM_, *bla*_CTX−M_	24	4
*Proteus mirabilis* T215	Blue shark	*qacEΔ1, bla*_CTX−M_, *aadA1*	32	16
*Listeria innocua* 4C21	Squid	*bla*_PSE_	12	4

*MIC, minimum inhibitory concentration (in mM)*.

### Genetic determinants of biocide tolerance and antibiotic resistance

Results obtained on the genetic determinants of resistance for the selected isolates are shown in Table 3. Twenty seven out of the 30 isolates tested positive for at least one of the genetic determinants studied. The only QAC resistance determinant detected was *qacE*Δ*1*. It was found in three isolates of *A. calcoaceticus* from salmon and in one *Proteus mirabilis* isolate from blue shark. The genetic determinant for sulfonamide resistance *sul1* was detected in 13 isolates, two of which also tested positive for *sul2*. In addition, three *A. calcoaceticus* isolates positive for *sul1* also were positive for *qacE*Δ*1*. However, PCR experiments using a qacEF primer and a sulR primer did not yield any amplification, suggesting that both genetic determinants were not physically close as in class I integrons.

Among the β-lactamase genes tested, *bla*_TEM_ and *bla*_CTX−M_ were the most frequent. Of the five isolates positive for *bla*_TEM_, three belonged to genus *Acinetobacter* isolated from salmon, one to *Aeromonas* and one to *Pseudmonas*. Five isolates tested positive for *bla*_CTX−M_: *Pseudomonas* (3), *Acinetobacter* and *Proteus*. The genetic determinant *bla*_PSE_ was detected in three isolates, including two *Pseudomonas* and the *L. innocua* isolate obtained from squid. Furthermore, *bla*_IMP_ and *bla*_NDM−1_ were detected in one *Pseudomonas* isolate each, both from anchovies. The remaining β-lactamase genes investigated were not detected.

The phenicol resistance determinant *floR* was detected in five isolates (all of them belonging to genus *Pseudomonas*) from different sources: anchovies, black mackerel and sea bass. By contrast, *cmlA* was not detected in any isolate. The aminoglycoside resistance determinant *aadA1* was detected in six isolates, five of which belonged to genus *Pseudomonas* and one to *Proteus*, while *aac(6*′*)-Ib* was detected in five isolates (including three from genus *Acinetobacter* isolated from salmon, one *Shewanella* and one *Pseudomonas*).

### Resistance to copper and zinc

A 26.60% of the 30 isolates tested were able to grow in the presence of 24 mM CuSO_4_, and still 23.30% were able to grow at 32 mM of the copper salt (Table 3). Regarding zinc tolerance, a 46.60% of the isolates were able to grow in the presence of 8 mM ZnCl_2_. However, all isolates tested were inhibited by 16 mM ZnCl_2_.

Study of the genetic determinants for metal resistance (Table 3) yielded positive results for the plasmid-borne multicopper oxidase gene *pcoA/copA* (36.66% of selected isolates) and the DNA binding repressor protein gene *pcoR* (6.66%), and for the zinc-chromate resistance gene *chrB* (13.33%). The remaining metal resistance genes investigated were not detected.

## Discussion

In the present study, biocide tolerance and antibiotic resistance were detected among bacteria isolated from seafoods. Biocides are used for many different purposes, including health care products and in disinfection processes in the food industry. For example, benzalkonium chloride is used for water treatment, general site disinfection, fish parasite removal, and prevention of infectious disease in fish and shellfish. As a result, large amounts of biocides arrive to waters. The impact of triclosan on aquatic bacterial communities has been described (Dann and Hontela, 2011; McNamara et al., 2014). Previous contact with biocides as well as natural background resistance could explain the biocide tolerances observed in the present study. It is also worth noting that there were positive correlations not only for tolerance to biocides of the same chemical group but also between biocides from different groups. However, there were differences between polyguanides, since poly-(hexamethylen guanidinium) hydrochloride showed positive correlation with several other biocides while chlorhexidine did not. These results could be explained by differences in chemical formula, mechanisms of adaptation (including intrinsic resistance), and also by the development of specific mechanisms of tolerance upon exposure to multiple biocides.

A relatively high percentage of isolates were resistant to at least one biocide and at least one antibiotic, and there were significant (*P* < 0.05) positive correlations between biocide tolerance and antibiotic resistance. These results reinforce the general concern that the use of biocides may co-select for antibiotic resistance (SCENIHR, 2009; Ortega-Morente et al., 2013; Wales and Davies, 2015). Cross-resistance between antibiotics and biocides and between different biocides has been reported for different bacteria, like for example *Pseudomonas aeruginosa* (Lambert et al., 2001; Lavilla Lerma et al., 2015). Furthermore, previous studies have shown that adaptation to biocides by repeated exposure results in an increased resistance to antibiotics (Gadea et al., 2016, 2017a). It is worth noting that there were also some positive correlations between tolerance/resistance to biocides, antibiotics, and other antimicrobials. For example, the phenolic biocide triclosan showed positive correlation with a lower sensitivity to the phenolic compound thymol (but not with carvacrol), and the antibiotic ampicillin also showed positive correlation with thymol. Previous studies have shown that exposure to plant essential oils (which are rich in phenolic compounds) such as pine oil resulted in the selection of mutants with deregulated *mar* operon that had decreased susceptibility to a range of antimicrobial compounds (including antibiotics and biocides) as a consequence of reduced cell permeability and increased efflux pump activity (Moken et al., 1997; Ortega-Morente et al., 2013). It is worth mentioning that the chemical preservatives sodium lactate and trisodium phosphate only showed moderate positive correlations with the antibiotics ampicillin and imipenem, but not with biocides. A recent study indicated that bacteria adapted to quaternary ammonium compounds under laboratory conditions showed a generalized increased tolerance to preservatives (such as 4-hydroxybenzoic acid, thyme, and clove oil, sodium, and potassium nitrates, potassium sorbate), while the opposite was observed in the case of triclosan (Gadea et al., 2017b).

Sulfonamides potentiated with trimethoprim or ormethoprim and florfenicol are some of the antibiotics commonly used in aquaculture (Hernández Serrano, 2005). In the present study, the sulfonamide resistance *sul1* gene was the genetic determinant detected most frequently. Sulfonamide resistance is most often linked to Class I integrons. These mobile genetic elements tend to accumulate different antibiotic resistance genes and also biocide tolerance genes like *qacE*Δ*1*. However, *qacE*Δ*1* was detected in combination with *sul1* only in the *A. calcoaceticus* isolates, and PCR amplification with a forward primer for *qacE*Δ*1* and a reverse primer for *sul1* did not suggest a close physical location of the two genes unless they were in opposite direction. Class 1 resistance integrons are located on mobile elements like transposons and plasmids and are widely distributed among clinical strains and also in environmental isolates, and play an important role as reservoirs of antimicrobial resistance genes (L'Abée-Lund and Sørum, 2001; Stokes and Gillings, 2011; Koczura et al., 2013, 2014). Remarkably, one study carried out at aquaculture facilities in the northern Baltic Sea (Finland) reported that antibiotic resistance genes for sulfonamides (*sul1* and *sul2*) and trimethoprim (*dfrA1*) and an integrase gene for a class 1 integron (*intI1*) persisted in sediments below fish farms at very low antibiotic concentrations during a 6-year observation period (Muziasari et al., 2014). Presumably, antimicrobial resistance genes could spread in marine sediments to other bacteria that colonize non-aquaculture fish and from these to seafood processing environments. This could explain the finding of *sul1* in bacteria isolated in the present study from fish like sardines, anchovies, Athlantic horse mackerel, sea bass, gilthead seabream, and salmon, or from squid. Furthermore, in a number of cases, bacterial isolates carrying *sul1* also tested positive for the florfenicol resistance gene *floR*, which can also be associated with Class 1 integrons (Toleman et al., 2007; Lin et al., 2016).

Aquaculture heavily depends on the use of antibiotics (Hernández Serrano, 2005), and several studies have reported on antimicrobial resistance in bacteria from aquaculture ecosystems (e.g., Shah et al., 2014; Huang et al., 2015; Xiong et al., 2015; Watts et al., 2017). Remarkably, one of the two isolates from the present study with broadest spectra of antimicrobial resistance (identified as *A. oleivorans*) was isolated from prawns grown in aquaculture. The *A. oleivorans* strain from the present study was resistant to 10 antimicrobials and tested positive for the genetic determinants *bla*_TEM_ and *bla*_CTX−M_. Since the prawns were boiled and frozen and then sold unfrozen over the counter on ice, there is a possibility that this strain arrived to the food by cross contamination during handling. Boiled prawns are ready to eat, therefore the bacteria with multiple antibiotic resistance together with their antibiotic resistance genes could be passed from prawns to humans. Acinetobacters are inhabitants of soil and water in addition to being opportunistic pathogens for humans, and the human-pathogenic strains are known to exhibit both intrinsic and acquired resistance to a wide variety of antimicrobials (Doughari et al., 2011). It is also worth noting that the remaining acinetobacters detected in the present study (all of them identified as *A. oleivorans*) were isolated from raw salmon slices, also grown in aquaculture. They were resistant to a lower number of antibiotics (4 and 6), but all of them tested positive for the genetic determinants *qacE*Δ*1, sul1* and *aac(6*′*)-Ib*, and two also were positive for the extended-spectrum β-lactamase gene *bla*_TEM_. *A. oleivorans* was described as a diesel-oil and n-hexadecane-degrading bacterium isolated from a rice paddy (Kang et al., 2011). So far, antibiotic resistance of non-pathogenic *Acinetobacter* species has been weakly explored, but results from the present study suggest that they could be an important reservoir of antimicrobial resistance traits.

A 14.94% of the 87 isolates from the present study were resistant to imipenem. Carbapenems were the last β-lactams retaining nearuniversal anti-Gram-negative activity, but carbapenemase genes are spreading, conferring resistance (Nordmann et al., 2011). Furthermore, among the metallo-β-lactamases investigated in the present study, the imipenemase (IMP), and the New Delhi metallo-β-lactamase NDM-1 gene were detected in two isolates, both belonging to *Pseudomonas*. Several metallo-β-lactamases encoded by mobile DNA have emerged in important Gram-negative pathogens (Cornaglia et al., 2011). NDM has been reported mainly in *Klebsiella pneumoniae* and *E. coli*, but it has also been found in a variety of other members of the *Enterobacteriaceae*, including *Acinetobacter* spp., in *Pseudomonas* spp., and in *Vibrio cholerae* (Poirel et al., 2011; Walsh et al., 2011; Mataseje et al., 2016). In *Pseudomonas aeruginosa*, the chromosomal *bla*_NDM−1_ gene has been reported to be located within a Class 1 integron bearing insertion sequence (IS) common region 1 (IS*CR1*) in a Tn*402*-like structure (Janvier et al., 2013; Jovcic et al., 2013). Remarkably, the integron also contained the QAC resistance determinant *qacE*Δ*1* and the sulfonamide resistance gene *sul1*. A similar gene array has been reported for the *bla*_NDM−1_ regions from *E. coli* transferable plasmid pNDM15-1078 (Mataseje et al., 2016). Furthermore, *bla*_NDM−1_ frequently appears in association with *ble*_MBL_ gene that confers resistance to bleomycin, a glycopeptide antibiotic that is naturally produced by *Streptomyces verticillus*. It is possible that bleomycin-like molecules contribute to selective pressure, leading to the further spread of NDM producers in the environment (Mataseje et al., 2016).

All *Aeromonas* isolates from the present study were resistant to chlorhexidine, ampicillin and imipenem. In addition two of them tested positive for *sul1* and *bla*_TEM_ and one also was positive for *bla*_CTX−M_. *Aeromonas* are widely distributed in aquatic environments (Holmes et al., 1996). The genus includes species pathogenic for fish (like *A. salmonicida* and others) and humans. In one study on ampicillin-resistant isolates from estuarine waters, Henriques et al. (2006) detected the presence of the integrase gene (along with other genes associated with Class I integrons) in *Aeromonas* strains. The authors also detected the presence of β-lactamase genes *bla*_TEM_, *bla*_SHV_, *bla*_CphA_, and *bla*_OXA−B_ in *Aeromonas* strains. Remarkably, the *bla*_OXA−B_ detected in *Aeromonas* sp. and *A. hydrophila* was associated with Class I integrons. Another study on *Aeromonas* isolated from nine freshwater trout farms in Australia reported the presence of *sul1* together with other antibiotic resistance genes typically associated with Class I integrons (Ndi and Barton, 2011). However, the β-lactamase genes investigated (*bla*_TEM_ and *bla*_SHV_) were not detected. Further studies should be carried out in order to determine the possible association of the β-lactamase genes detected in the *Aeromonas* isolates from present study with Class I integrons.

In the present study, one isolate resistant to cefotaxime, ceftazidime, streptomycin and nalidixic acid was identified as *Listeria innocua*. This isolate tested positive for the β-lactamase resistance gene *bla*_PSE_. Previous studies have reported on antimicrobial resistance in *Listeria* spp. isolated from raw fish and open-air fish market environment (Jamali et al., 2015). The authors reported a high resistance of *L. monocytogenes* to tetracycline and penicillin G in agreement with other studies (Rodas-Suárez et al., 2006; Fallah et al., 2013). Another work reported a high resistance level to ampicillin, cefotaxime (100%), and penicillin (57%) in seafood isolates of *L. monocytogenes* (Abdollahzadeh et al., 2016). We could speculate that the *L. innocua* isolated in the present study from squid originated from the seafood handling and processing environment. Although this is a non-pathogenic species, the results would suggest that different species of *Listeria* in seafood processing environments can act as reservoirs for antimicrobial resistance traits.

Exposure to low concentrations of antibiotics, disinfectants, chemical pollutants, and metals can act as a selective force leading to resistance processes among indigenous bacterial populations (Martinez, 2009). There is a concern that exposure to metals may co-select for antibiotic resistance. In the present study, isolates capable of growing at high concentrations of copper sulfate, and zinc chloride included representatives of *Pseudomonas, Aeromonas, Acinetobacter*, and *Proteus*. The observed metal tolerance could be due to direct exposure to these metals in the environment. Furthermore, the multicopper oxidase gene *pcoA/copA* was detected in several *Pseudomonas* strains that were also positive for different antibiotic resistance genes (*bla*_CTX−M_, *bla*_TEM_, *bla*_PSE_, *bla*_NDM−1_, *bla*_IMP_, *aadA1, aac(6*′*)-Ib, sul1, sul2, floR*). The copper-inducible system *copABCDRS* was first described within the plasmid pPT23D in *P. syringae* (Cha and Cooksey, 1991) and is homologous to the *pco* system found on the conjugative plasmid pRJ1004 from *E. coli*. In addition to the plasmid-borne system, chromosomal P-type ATPases are responsible for conferring tolerance to metals like copper, zinc, cobalt, chromium, and cadmium (Teitzel and Parsek, 2003). As indicated by Berendonk et al. (2015), selective pressures present in natural environments such as rivers and lakes as a result of human practices are leading to the occurrence of an increasing number of multi-drug resistant environmental *Pseudomonas* isolates (Berendonk et al., 2015). Nevertheless, in the study of the resistome of *P. aeruginosa* E67, an epiphytic isolate from a metal-contaminated estuary, physical links between metal and antibiotic resistance genes were not identified, suggesting a predominance of cross-resistance associated with multidrug efflux pumps (Teixeira et al., 2016). Bacterial multidrug efflux pumps can accommodate a variety of antimicrobials, including dyes, antibiotics, and biocides (Poole, 2007). Nevertheless, a possible association of plasmidic *pcoA/copA* with antibiotic resistance genes in *Pseudomonas* deserves to be further investigated.

In conclusion results from the present study clearly indicate that bacteria from seafoods carry resistance traits against diverse antimicrobials. The possible role of these bacteria in spread of antimicrobial resistance through the food chain deserves further investigation. It is also important to find alternative ways to manage antimicrobial resistance. Biological methods such as the use of probiotics (Tan et al., 2016; Banerjee and Ray, 2017) or the application of bacteriophages (Letchumanan et al., 2016; Torres-Barceló et al., 2016; Parmar et al., 2017) could be promising alternatives to classical antimicrobials.

## Author contributions

JR carried out seafood sampling, microbiological analysis and PCR investigation of genetic determinants of resistance. MG participated in planning and supervision of the experimental work and contributed with strain identification and preparation of PCR experiments. RP contributed with data analysis and preparation of graphical material for the manuscript. RL and AG carried out global analysis of the results and wrote the manuscript.

### Conflict of interest statement

The authors declare that the research was conducted in the absence of any commercial or financial relationships that could be construed as a potential conflict of interest.
